# Yellow discoloration of the fingers due to golimumab with dermoscopic features: A brief literature review

**DOI:** 10.1111/jocd.16391

**Published:** 2024-06-17

**Authors:** Erhan Ayhan, Mücahit Yaşa

**Affiliations:** ^1^ Department of Dermatology Dicle University Faculty of Medicine Diyarbakır Turkey

## ETHICS STATEMENT

Written informed consent was obtained from the patient.

To the editor,

Golimumab is a human monoclonal antibody specific for TNF‐α that specifically binds to transmembrane and soluble TNF‐α receptors and blocks their activity. It is approved by the FDA for the treatment of moderate to severe rheumatoid arthritis, psoriatic arthritis and ankylosing spondylitis. Common side effects include superficial fungal infections, bacterial infections, urticaria, oral pain and ulcers, injection site reactions. In this article, we report a male patient who developed yellow discoloration of the fingers after golimumab, which is stated to be rarely seen in the prescribing information but there is no report in the literature.

A 30‐year‐old male patient presented to our outpatient clinic with yellowish spots on both fingers. It was learned from his history that he had been under treatment with golimumab for ankylosing spondylitis for about 6 years. However, 15 days after the last dose, he stated that he had painless, non‐pruritic yellow discoloration on his fingers and noticed that it decreased during the day. The patient had no history of dietary changes, diabetes mellitus or any other drug use that could cause discoloration of the hands. On dermatologic examination, yellowish‐orange discoloration was observed on both fingers, especially on the thumb and index finger tips (Figure 1). No color abnormality was observed on the feet or the rest of the body. Dermoscopic examination revealed that the skin surface of the thumb was completely diffusely yellowish‐orange, but some areas showed brown pigment concentration in the sulci (Figure 2). Laboratory parameters including blood carotene, lipid and bilirubin levels were within normal limits. Since the patient had no history of medication use other than golimumab, normal laboratory values, no systemic disease and no excessive consumption of foods that may cause yellow discoloration in the diet, the yellow discoloration of the fingers was considered to be due to golimumab.

**FIGURE 1:**
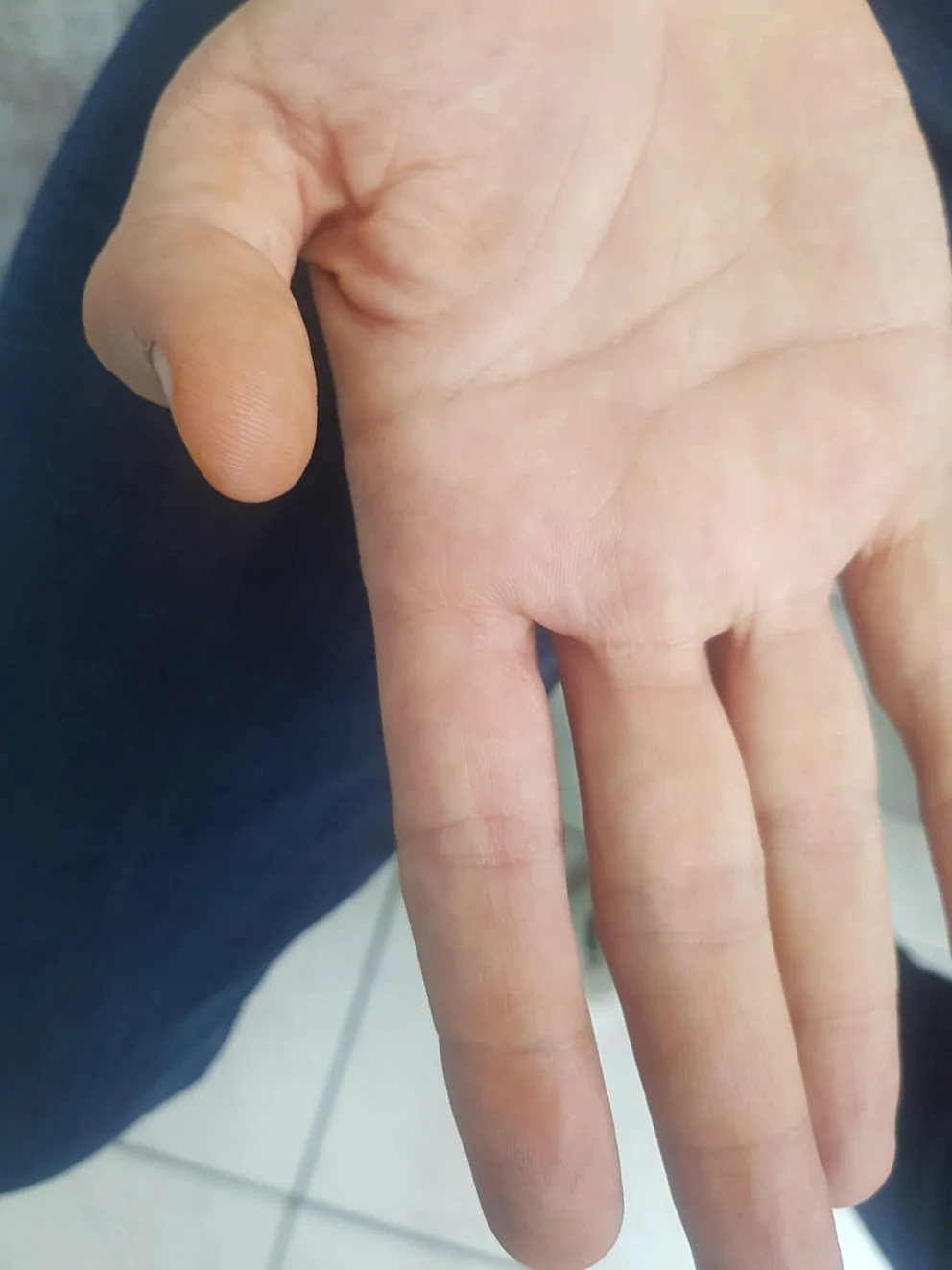
The patient has yellow discoloration of the right hand, especially the thumb and index finger.

**FIGURE 2:**
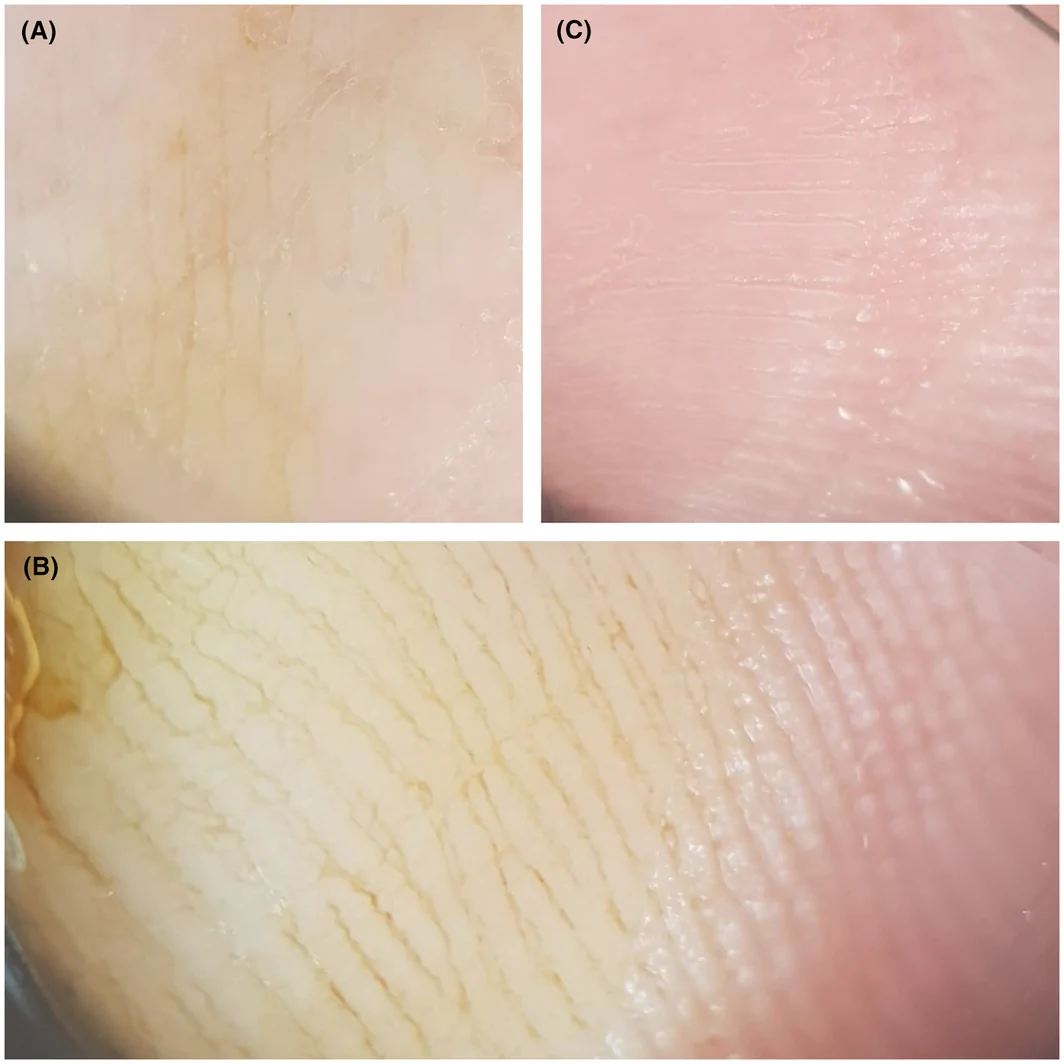
Dermoscopy shows a (A) yellowish background on the thumb and (B) increased brownish pigment density in the sulci. (C) The patient's dermoscopic image 4 months later shows that there is no yellow discoloration.

Yellow discoloration of the skin may be associated with diabetes mellitus, carotenemia, hypothyroidism, liver disease and kidney disease.^1^ Hyperlipoproteinemia type 2 A, which is among the causes of hypercholesterolemia, can also cause yellow discoloration of the hands and feet without affecting the nails.^2^ However, carotenemia is the most common cause of yellow discoloration of the hands and feet.^1^, ^3^ Many green, yellow and orange fruits and vegetables such as carrots, sweet potatoes and squash contain carotene.^3^ It is important to recognize carotenemia and not confuse it with jaundice. The presence of clear sclera is an important clinical sign that can help distinguish carotenemia from jaundice. Normal bilirubin levels and high levels of carotene in the blood can confirm the diagnosis.^3^

The main drugs thought to cause skin pigmentation are nonsteroidal anti‐inflammatory drugs, antimalarials, amiodarone, cytotoxic drugs, tetracyclines, heavy metals, and psychotropic drugs. The clinical features are very variable according to the triggering molecule and have a wide range of patterns and colors, sometimes more or less reminiscent of the offending drug. Among these drugs, quinacrine, and mepacrine used in the treatment of lupus erythematosus cause yellow discoloration of the whole body.^4^, ^5^ However, they usually cause yellow discoloration of the whole body at a level that can mimic jaundice.^4^ Yellow discoloration of the hands and feet was also reported in a patient using disulfiram.^6^ However, OTC products used for acne can also cause yellow discoloration on the hands for a long period of 2–3 months.^7^

Although yellow discoloration of the fingers and toes is listed as a rare side effect in the prescribing information of golimumab, its absence in the literature reduces its awareness. Although it is not clear whether the yellow discoloration of the hand in our case was due to contact of golimumab to the hands or a systemic side effect, we consider it important that this case is reported in the literature.

## FUNDING INFORMATION

None.

## CONFLICT OF INTEREST STATEMENT

All authors declare that they have no conflicts of interest.

## Data Availability

The data that support the findings of this study are available from the corresponding author upon reasonable request.
